# Manipulation and Applications of Hotspots in Nanostructured Surfaces and Thin Films

**DOI:** 10.3390/nano10091667

**Published:** 2020-08-26

**Authors:** Xiaoyu Zhao, Jiahong Wen, Aonan Zhu, Mingyu Cheng, Qi Zhu, Xiaolong Zhang, Yaxin Wang, Yongjun Zhang

**Affiliations:** 1 Innovative Center for Advanced Materials (ICAM), School of Material and Environmental Engineering, Hangzhou Dianzi University, Hangzhou 310018, China; zhaoxy@hdu.edu.cn (X.Z.); wangyaxin1010@126.com (Y.W.); 2 National Laboratory of Solid State Microstructures, Nanjing University, Nanjing 210093, China; 3 The College of Electronics and Information, Hangzhou Dianzi University, Hangzhou 310018, China; 4 Key Laboratory of Functional Materials Physics and Chemistry, Ministry of Education, College of Physics, Jilin Normal University, Changchun 130103, China; aonanzhu@126.com (A.Z.); chengmingyu0531@163.com (M.C.); qizhu12300@163.com (Q.Z.)

**Keywords:** nanostructured surfaces and thin films, physical vapor deposition, nanosphere lithography, manipulation and applications of hotspots

## Abstract

The synthesis of nanostructured surfaces and thin films has potential applications in the field of plasmonics, including plasmon sensors, plasmon-enhanced molecular spectroscopy (PEMS), plasmon-mediated chemical reactions (PMCRs), and so on. In this article, we review various nanostructured surfaces and thin films obtained by the combination of nanosphere lithography (NSL) and physical vapor deposition. Plasmonic nanostructured surfaces and thin films can be fabricated by controlling the deposition process, etching time, transfer, fabrication routes, and their combination steps, which manipulate the formation, distribution, and evolution of hotspots. Based on these hotspots, PEMS and PMCRs can be achieved. This is especially significant for the early diagnosis of hepatocellular carcinoma (HCC) based on surface-enhanced Raman scattering (SERS) and controlling the growth locations of Ag nanoparticles (AgNPs) in nanostructured surfaces and thin films, which is expected to enhance the optical and sensing performance.

## 1. Introduction

The synthesis of nanostructured surfaces and thin films using physical vapor deposition, such as pulsed laser deposition, magnetron sputtering, thermal evaporation, e-beam evaporation, among others, plays a key role in the development of a variety of applications in nanoplasmonics, nanoscale photovoltaic devices, nanogenerators, flexible or nanobiological sensors, and so on [1,2,3,4,5,6,7,8]. For devices based on nanostructured surfaces and thin films, diverse high-fidelity geometry is important for the performance of the devices in practical applications. Reliable artificial nanopatterned surfaces and thin films are fabricated by advanced lithographic methods, including electron-beam lithography (EBL), photolithography, soft lithography, nanosphere lithography (NSL), and many others [9,10,11,12]. For example, the combined processes of EBL, metal deposition, and liftoff are utilized to obtain patterned metallic structures on a scale of tens of nanometers to submillimeter [12]. However, the multiple wet processes in EBL-based methodology is extremely time-consuming and may introduce additional contaminations on the nanostructured surfaces, which may have non-negligible effects on the quality of nanostructured surfaces and thus degrade the performance of the devices. More importantly, it significantly hinders the direct applicability of the devices, especially in the field of nanostructure-based plasmonics, generators, sensors, and so on. Though many efforts have been made to prepare nanostructured surfaces and thin films by EBL-based methodology, challenges remain in the manipulation of hotspots that are usually observed in the sub-10 nm metallic nanogaps, where the energy is localized to subwavelength dimensions due to the design of the nanostructured surfaces and thin films.

Compared to the EBL-based methodology, the NSL-based approach has attracted much attention. This method uses self-assembled polystyrene (PS) colloid sphere arrays as ordered templates/masks to manipulate hotspots in nanostructured surfaces and thin films. The method is rapid, simple, low-cost, practical, and produces no pollution [13]. Various nanostructured surfaces and thin films can be achieved by the combination of NSL and physical vapor deposition, such as periodic nanocaps [14,15,16,17,18], nanotriangles [19,20,21,22], nanobowls [23,24,25], nanorings [26,27,28], nanopillars [29,30], nanocones [31,32,33], and other complex nanostructured surfaces and thin films, including nanohoneycomb, bridged knobby units, nanoparticle cluster-in-bowl arrays, and so on [2,25,34,35,36,37,38,39,40]. These architectural designs of nanostructured surfaces and thin films can be obtained by controlling a series of deposition processes (the deposition time, angle, distance, and so on), PS colloid sphere etching, transfer, and their combination steps, which manipulate the formation, distribution, and evolution of hotspots and have significant implications in broad applications [41,42,43,44,45,46,47,48,49,50,51,52,53,54,55]. Based on these manipulation of hotspots in nanostructure-based surfaces and thin films, plasmon-enhanced molecular spectroscopy (PEMS) and plasmon-mediated chemical reactions (PMCRs) can be controlled, which is expected to enhance the optical and sensing performance.

In this article, the design and synthesis of nanostructured surfaces and thin films with various hybridization of nanoshape arrays are discussed in detail, including large-area periodic nanohoneycomb, nanocap star, nanoring nanoparticle, bridged knobby units, and three-dimensional (3D) nanopillar cap arrays. The formation, distribution, and evolution of hotspots in these nanostructured surfaces and thin films are controlled, which has potential applications in PEMS and PMCRs. Hopefully, this article will inspire more ingenious designs of nanostructured surfaces and thin films using the NSL technique to manipulate hotspots, which is expected to enhance the optical and sensing performance.

## 2. Experimental Section

### 2.1. NSL Technique and Physical Vapor Deposition Technique

The NSL technique originates from self-assembled monolayer nanospheres being used as a mask to achieve large-area surface-patterned nanostructures, also known as “natural lithography” [49]. The nanosphere particles are arranged in an ordered array by spin coating, Langmuir–Blodgett technique, electrophoretic deposition, micropropulsive injection (MPI) method, and so on [56,57,58]. Then, defect-free PS colloid sphere arrays from single layer (SL) to multilayer (ML) are fabricated, which greatly extends the application of “natural lithography” and is called NSL [21,27,50,59]. Due to developments over the past several decades, the NSL technique has been recognized as an effective way to fabricate large-scale ordered nanostructured surfaces and thin films with various nanopatterns [34,42,60]. Normally, NSL have three main processes. First, SL or ML closely packed PS colloid sphere arrays are prepared by the self-assembly method [27,28,56,57,58,61]. Briefly, PS colloid sphere particles are dispersed in alcoholic solution, which is slowly dripped on the surface of a Si wafer. Then, the Si wafer is slowly immersed into the container filled with deionized water. At the interface between the PS colloid sphere particles and deionized water, the PS colloid spheres start to form an unordered monolayer. After that, the monolayer is driven into a highly ordered array by interactions including van der Waals forces, steric repulsions, and Coulombic repulsions [57]. Then, the highly ordered PS colloid sphere array is picked up by the hydrophilic property substrate. The detailed preparation process of large-scale ordered PS colloid sphere arrays is reported in our previous works [9,13,17,18]. Then, the size, nanogaps, and surface morphology of the as-prepared PS colloid sphere arrays is modified by physical and chemical method, which serves as a template [29,30,37,38,39,53]. Finally, various materials, including metal, metal oxides, polymers, and so on, are deposited on the ordered PS colloid sphere array templates by physical vapor deposition technique (for example, pulsed laser deposition, magnetron sputtering, thermal evaporation, e-beam evaporation), which make the nanostructured surfaces and thin films more functional.

### 2.2. Design of Nanostructured Surfaces and Thin Films and Manipulation of Hotspots

Based on the combination of the as-prepared PS colloid sphere array templates and physical vapor deposition, various nanostructured surfaces and thin films can be designed by adjusting the fabrication routes and deposition parameters. Under this strategy, PS colloid sphere arrays with a fixed diameter (e.g., 100, 200, 500, 1000 nm) are selected as templates, and the etching, transfer, rotation, co-sputtering, glancing angle sputtering, single- or multilayer deposition, or their combinations is performed to control the shape of the PS colloid spheres, nanogaps between neighboring PS colloid spheres, the thickness of films, the surface morphology of films, and other parameters of the complex structures. The variety of nanostructured surfaces and thin films can be expanded, obtaining a set of novel nanopatterned arrays by the corresponding control strategy. Several types of novel nanopatterned arrays, including nanohoneycomb, nanocap star, nanoring nanoparticle, bridged knobby units, nanopillar cap, and other hybrid nanostructure arrays, are described in detail in the next section.

At the nanostructured surfaces of some noble metals (e.g., Au, Ag, etc.), the coherent oscillations of the conduction electrons can be driven by light, which will cause localized surface plasmon resonances (LSPRs). Usually, the electromagnetic (EM) field is enormously enhanced in the region of LSPRs, which is called “hotspots”. As is well known, these hotspots are usually observed in the sharp tips or corners, sub-10 nm metallic nanogaps, and so on. Moreover, the formation, distribution, and evolution of hotspots are very sensitive to the material, composition, and surrounding dielectric environment of the nanostructures. Therefore, the manipulation of hotspots can be achieved by adjusting the aspects mentioned above. To confirm the formation, distribution, and evolution of hotspots, finite-difference time-domain (FDTD) software is used to simulate the EM field of the nanostructured surfaces and thin films, which has guiding significance on the design of nanostructured surfaces and thin films and the manipulation of hotspots.

### 2.3. Applications of Hotspots in PEMS and PMCRs

PEMS is a rapid and nondestructive spectroscopy technique for chemical detection, biosensing, catalysis, and so on. The technique relies on the high density of hotspots to significantly intensify surface-enhanced Raman scattering (SERS) signals, which is expected to achieve single-molecule detection. Based on the SERS spectroscopy technique, nanostructured surfaces and thin films with high density of hotspots as biomarker chips exhibit excellent performance for the specific detection of α-fetoprotein (AFP) and α-fetoprotein-L3 (AFP-L3), which are very promising for the detection of early hepatocellular carcinoma (HCC) markers [38,39].

In addition, based on manipulation of hotspots in nanostructured surfaces and thin films, PMCRs have attracted much attention. Active sites with selectively controlled chemical reactions at the nanometer level can be achieved by manipulating the formation, distribution, and evolution of hotspots in nanostructured surfaces and thin films, which is an easy method to obtain a wide variety of ordered nanostructures and is expected to enhance plasmonic performance.

## 3. Results and Discussion

### 3.1. Manipulation of Hotspots in Nanostructured Surfaces and Thin Films

**Figure 1:**
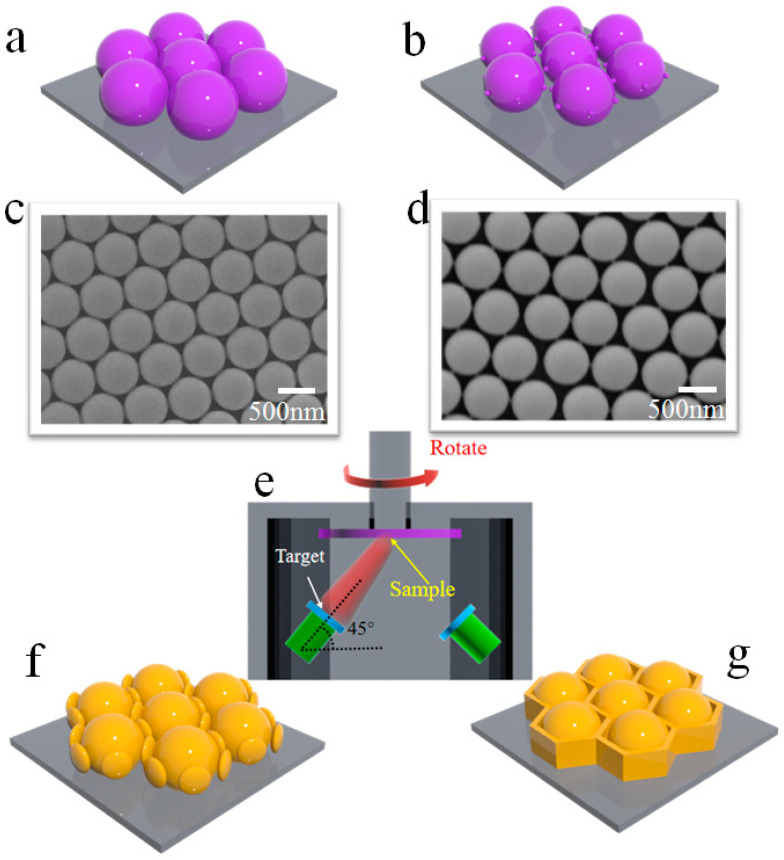
(**a**,**b**) Schematic diagram of hexagonally arranged polystyrene (PS) colloid sphere array and PS colloid sphere array with six tiny synaptic nanostructures after reactive ion etching (RIE) treatment, respectively. (**c**,**d**) SEM image of hexagonally arranged PS colloid sphere array before and after RIE treatment. (**e**) Schematic diagram of the magnetron sputtering chamber. (**f**,**g**) Desired honeycomb nanostructure achieved by controlling rotational speed and film sputtering time. Reproduced with permission from [38] American Chemical Society, 2019.

The architectural design and fabrication of multiscale nanostructured surfaces and thin films are carried out by the combination of NSL and magnetron sputtering deposition. An ordered PS colloid sphere array with a size of 500 nm is fabricated by the self-assembly process on Si wafer, which is shown in the schematic diagram in Figure 1a and the scanning electron microscopy (SEM) image in Figure 1c. Hexagonally arranged PS colloid sphere arrays with different sizes and layers can be obtained by a similar process. Using as-prepared PS colloid sphere arrays as a template, when thin films are deposited along the perpendicular direction, two basic nanostructures (nanocap array and triangular-shaped array) are formed, which is the simplest design. By accurately controlling the parameters of the ordered PS colloid sphere array and the processes of magnetron sputtering deposition, basic nanopatterned arrays can be expanded and reinvented to novel nanostructured surfaces. For example, a novel honeycomb nanostructured array can be prepared by selective reactive ion etching (RIE) and glancing angle sputtering with rotation. In the first step, the as-prepared monolayer PS colloid sphere array is etched for different times, which results in six tiny synaptic nanostructures per PS colloid sphere due to the shadow effect, as shown in the schematic diagram in Figure 1b and SEM image in Figure 1d. These tiny synaptic structures among the PS colloid sphere play a key role in the subsequent design of the honeycomb nanostructured array. Then, the Au film is glancing angle sputtered onto the PS colloid sphere array template with tiny synaptic nanostructures by magnetron sputtering (Figure 1e). During the film deposition, the evolution of a well-formed honeycomb nanostructure can be achieved by controlling the rotation speed of the PS colloid sphere array template and film sputtering time, as shown in Figure 1f,g.

**Figure 2:**
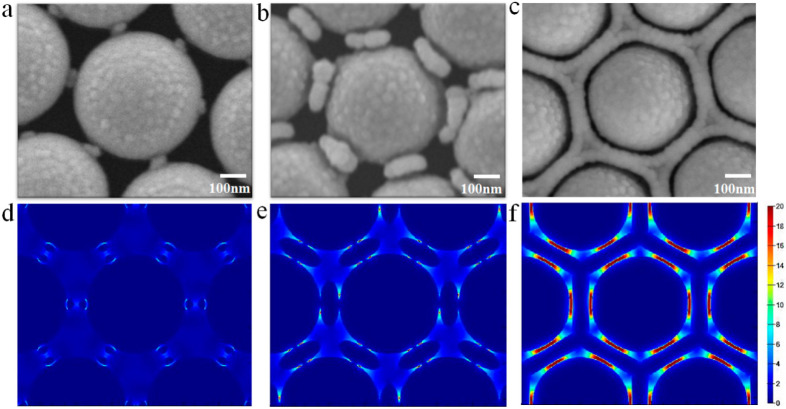
(**a**–**c**) SEM images of the process of honeycomb nanostructure formation with different deposition times (5, 20, and 40 min, respectively). (**d**–**f**) The finite-difference time-domain (FDTD) simulation for sectional views of the local EM field distribution in the process of honeycomb nanostructure formation. Reproduced with permission from [38] American Chemical Society, 2019.

The morphological features and distribution of hotspots of the honeycomb nanostructures are exactly controlled by RIE time, film sputtering time, and rotation speed during film deposition. To obtain a PS colloid sphere array with tiny synaptic nanostructures and suitable separation, the as-prepared PS colloid sphere array is etched for 120, 180, 240, or 300 s. To optimize the morphological features, we can increase the rotational speed of the etched PS colloid sphere array during the film deposition process (from 10 to 60 rpm), which promotes the rate of film deposition onto the tiny synaptic nanostructure. When the PS colloid sphere array is etched 180 s and the rotation speed is 60 rpm, the honeycomb nanostructure is gradually distinct. The evolution of the honeycomb nanostructured morphology depends on the film deposition time, as shown in Figure 2a–c. When the film deposition time is 5 min, the morphology of the etched PS colloid sphere surface and around the tiny synaptic nanostructures show slight changes (Figure 2a). When the film deposition is increased to 20 min, the synaptic nanostructures around each PS colloid sphere exhibit continuous growth (Figure 2b). When the film deposition time reaches 40 min, a satisfactory honeycomb nanostructure is achieved, where the nanogaps between the sidewalls and the nanocaps are sub-10 nm (Figure 2c). We know that hotspots usually localize in the sharp tips, corners, and sub-10 nm gaps of noble metallic nanostructures, where the coupling EM field is enormously enhanced. FDTD solutions (Lumerical Solutions Inc, Vancouver, BC, Canada) is utilized to simulate the formation, distribution, and evolution of the local EM field in nanostructured surfaces and thin films, where the relevant nanostructural parameters are extracted from the actual prepared patterned nanostructures. Figure 2d–f shows the EM intensity for three fabricated samples (Au film deposition times of 5, 20, and 40 min). The FDTD results indicate that the formation, distribution, and evolution of hotspots can be manipulated by changing the honeycomb nanostructured morphology. The local EM field of the synaptic parts is increased by promoting growth in tiny synaptic structures among the PS colloid sphere (Figure 2d). With an increase in the film deposition time, the hotspots appear and increase in the synaptic nanostructures, as shown in Figure 2e. It is obvious that the local EM field distribution in the nanogaps between the sidewalls and the nanocaps lead to the density of hotspots being predominantly enhanced in a satisfactory honeycomb nanostructure (Figure 2f).

**Figure 3:**
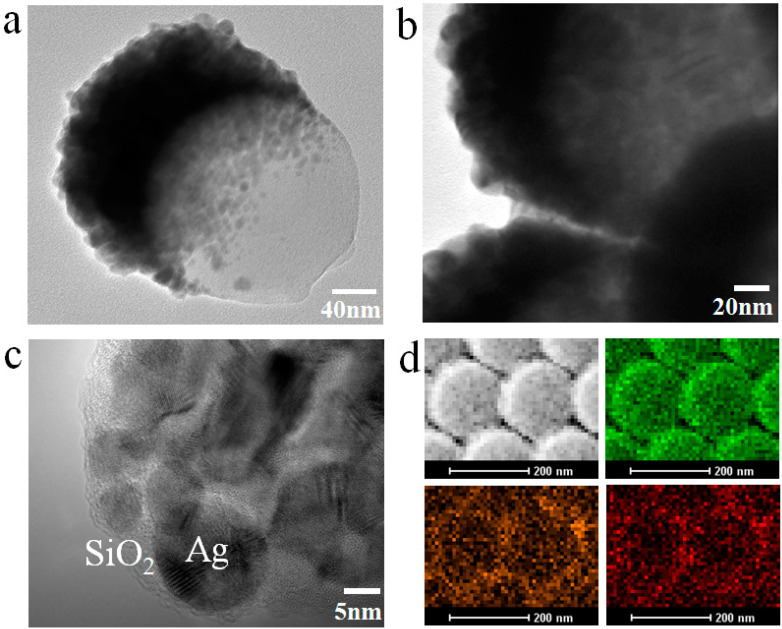
(**a**–**d**) TEM, HRTEM, and element analysis images of the SiO_2_−Ag nanocap. Reproduced with permission from [9] American Chemical Society, 2014.

In addition, according to the design requirements, various hybridized, complex, or novel nanostructured surfaces and thin films can be obtained by controlling the preparation strategy. Under a similar process, nanocap star, nanoring nanoparticle, and others nanostructured arrays were achieved in our previous reports [37]. In addition to the size and morphology of nanostructured arrays, the composition of nanostructured surfaces and thin films and the surrounding dielectric environment also play important roles in the manipulation of hotspots. Based on this strategy, noble metals and insulator composites are co-sputtered onto closely ordered PS colloid sphere (200 nm) arrays by magnetron sputtering system (ATC 1800-F, USA AJA). Taking co-deposition of Ag and SiO_2_ as an example, the SiO_2_-isolated Ag island (SiO_2_−Ag) nanocap forms on the PS colloid sphere, as shown in Figure 3a. The transmission electron microscopy (TEM) image shows that the size of the nanogaps between adjacent SiO_2_-isolated Ag nanocaps is under sub-10 nm (Figure 3b). The high-resolution transmission electron microscopy (HRTEM) image indicates that the thickness of the amorphous SiO_2_ and the size of Ag nanoparticles (AgNPs) are around 2−5 and 5−10 nm, respectively. These amorphous SiO_2_ and Ag nanoparticles are intertwined, which helps form nanoscaled surface roughness and more nanogaps (Figure 3c). The corresponding area element analysis mapping of SiO_2_−Ag nanocap arrays show that Ag, Si, and O elements are uniformly distributed in the nanocaps, as shown in Figure 3d.

**Figure 4:**
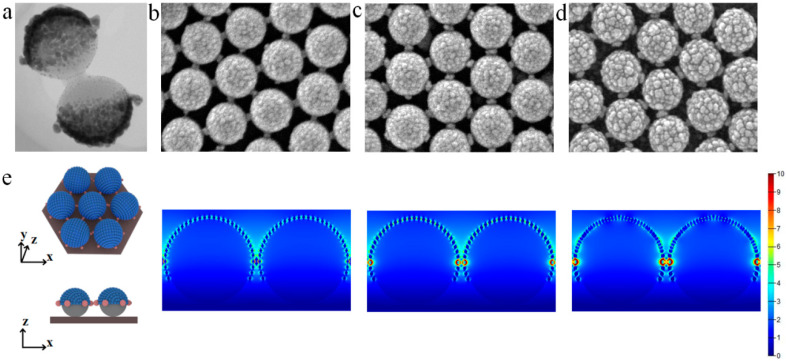
(**a**–**d**) TEM and SEM images of the SiO_2_−Ag film co-sputtered (5, 15, and 20 min) onto PS colloid sphere array after being etched for 60 s. (**e**) The idealized morphology and simulation results of SiO_2_−Ag nanocaps in the FDTD. Reproduced from [39] with permission from Elsevier, 2020.

When closely ordered PS colloid sphere arrays are etched, the diameter of PS colloid spheres decreases, and tiny synaptic nanostructures around each PS colloid sphere are observed. After SiO_2_−Ag film deposition, the SiO_2_−Ag nanocap is still formed on the smaller PS colloid spheres, as shown in Figure 4a. The film preferentially grows around the tiny synaptic nanostructures, which forms a bridge between adjacent nanocaps. With increasing deposition time, the surface roughness of the SiO_2_−Ag nanocaps increases, and the nanogaps between the units of bridged knobby units gradually decrease (Figure 4b–d). The results of FDTD simulations indicate that the hotspots where the EM field is coupling are mostly distributed on the surface of the SiO_2_-isolated Ag nanoparticles on the nanocaps and the bridges between nanocaps, as shown in Figure 4e. In addition, the trilayer or multilayer Ag/SiO_2_ composite shell or 3D pillar-cap arrays also significantly improve the enhancement of the EM field, which manipulates the distribution of hotspots [29,45,51]. The SiO_2_ addition not only immensely increases the surface roughness of the designed nanostructure surfaces and thin films but also improves the enhancement of the EM field at the nanogaps, which manipulates the formation and evolution of hotspots. The manipulation of hotspots in nanostructured surfaces and thin films has potential applications in the field of plasmonics, including SERS, biomarker chips, mediated chemical reactions, and so on.

### 3.2. Applications of Hotspots in Plasmonics

**Figure 5:**
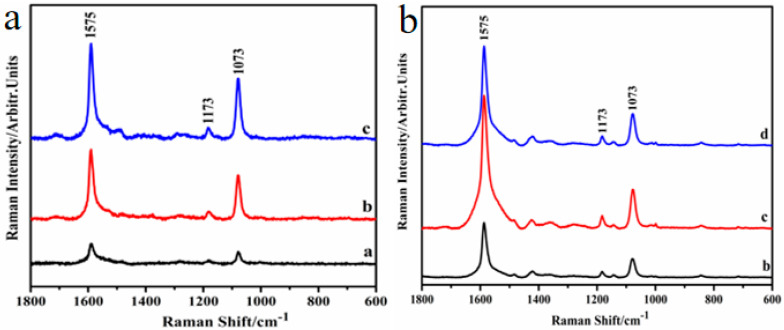
(**a**,**b**) The surface-enhanced Raman scattering (SERS) spectrum of 4-mercaptobenzoic acid (4-MBA) for the Au nanohoneycomb and SiO_2_−Ag nanocap arrays, which correspond to Figure 2a–c and Figure 4b–d, respectively. Reproduced with permission from [38] American Chemical Society, 2019; Reproduced with permission from [9] American Chemical Society, 2014.

PEMS and PMCRs are two important branches in plasmonics. PEMS includes fluorescence spectrum, infrared spectrum, and SERS. Among the three spectra, SERS is the most promising spectroscopy technique, which can achieve obviously enhanced Raman signals by hotspots in nanostructured surfaces and thin films. As mentioned above, Au nanohoneycomb and SiO_2_−Ag nanocap arrays show typical SERS spectra when 4-mercaptobenzoic acid (4-MBA) is used as a probe. Figure 5a–b show that SERS peaks at about 1575, 1073, and 1173 cm^−1^ are assigned to the aromatic ring vibrations and the C−H deformation vibration modes, respectively [37]. The SERS intensity of 4-MBA increases with the manipulation of hotspots in nanostructured surfaces and thin films and obtains the highest enhancement, which is in good agreement with the FDTD simulation.

**Figure 6:**
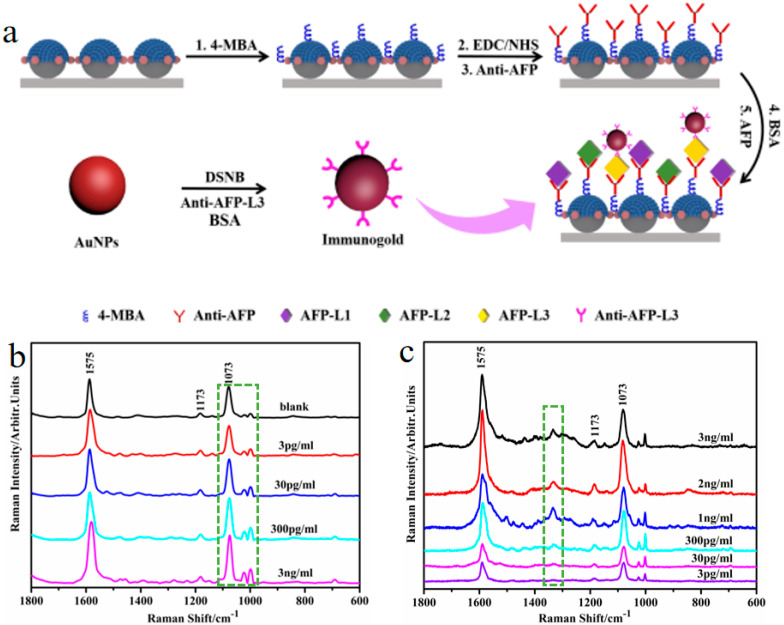
(**a**) The preparation schematic diagram of SiO_2_−Ag nanocap arrays as a biomarker chip. (**b**,**c**) The SERS spectrum of the biomarker chip for different α-fetoprotein (AFP) and α-fetoprotein-L3 (AFP-L3) concentrations, respectively. Reproduced from [39] with permission from Elsevier, 2020.

Based on the SERS technique, the biological and biomedical detection of some mortal diseases has been achieved by determining changes in the shift and intensity of the SERS signals. For instance, using SiO_2_−Ag nanocap arrays with high density of hotspots as biomarker chips, the early diagnosis for HCC can be detected based on the analysis of the shift in characteristic peaks of the probe molecule 4-MBA and AFP-L3. The whole preparation and immune process of biomarker chips for the detection of HCC is shown in Figure 6a. First, the biomarker chip, which is composed of SiO_2_−Ag nanocap arrays with bridges, is immersed in the 4-MBA, 1-(3-(dimethylamino)propyl)-3-ethylcarbodimide hydrochloride (EDC), and N-hydroxysuccinimide (NHS) solutions. Then, the 4-MBA-derived coupling agent is generated using EDC–NHS, which is used to bind the anti-AFP antibody. After the reaction, bovine serum albumin (BSA) is added into the mixed solution to block unconnected anti-AFP antibodies.

In addition, the anti-AFP is diluted and used as a blank contrast sample. Antigens with different concentrations (3, 30, 300, and 3000 pg/mL) are added and allowed to react with the biomarker chip in the centrifuge tubes. After the process, antibody-capturing chips are made and characterized by SERS. The SERS spectrum of 4-MBA connected with different AFP concentrations are shown in Figure 6b. When the concentration of the AFP increases, the peaks of 4-MBA around 1073 cm^−1^ shifts to the left and the intensity of the peaks at 998 cm^−1^ are enhanced, as shown by the dashed frame in Figure 6b. The changes in the two peaks confirm the success of the MBA-based antibody absorption. Subsequently, the preparation of immunogold and immunological recognition are implemented to detect HCC by analyzing the ratio of AFP-L3 to total AFP. For analyzing AFP-L3, 5,5′-dithiobis (succinimidyl-2-nitrobenzoate) (DSNB) is used as a probe between the antibody and the AuNPs. Colloidal gold is fabricated by the Lee and Meisel approach, which is added to the DSNB acetonitrile solution. Next, the anti-AFP-L3 is put in and stored at room temperature. After the reaction, BSA and borate buffer are added into the solution for later use. Finally, the biomarker chip is formed after being immersed in solutions of different concentrations (3, 2, 1 ng/mL and 300, 30, 3 pg/mL) to ensure sufficient immunological recognition. Figure 6c shows the SERS spectrum of the immunogold-decorated biomarker chip with different AFP-L3 concentrations. The SERS peak at 1331 cm^−1^ is used as a characteristic immunogold signal in the dashed frame in Figure 6c, which reflects the degree of coupling between AuNPs. With the methods mentioned above, the detection limits for AFP and AFP-L3 are below 3 pg/mL, which proves that the designed nanostructured surfaces and thin films with ordered hotspots is of great significance for the early detection of HCC, clinical application, and SERS immune detection [38,39].

**Figure 7:**
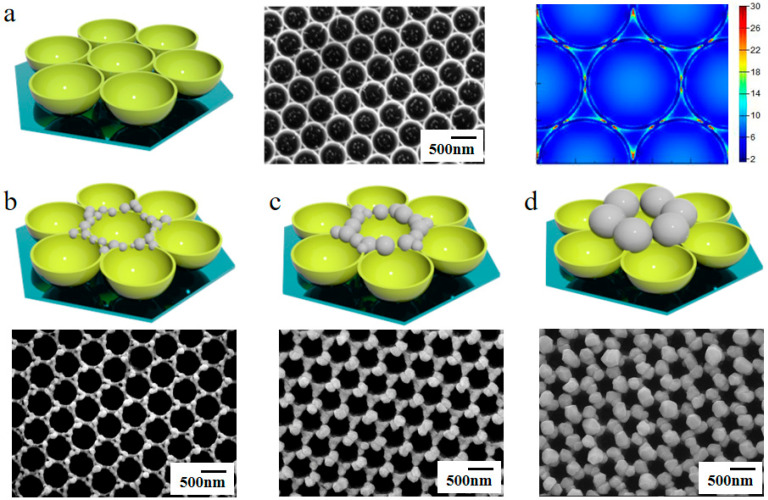
(**a**) SEM image and the distribution of hotspots for the Au nanobowl arrays. (**b**–**d**) SEM images of the Au nanobowl arrays with Ag nanoparticles (AgNPs) accurately grown at the defined hotspot location. Reproduced from [13] with permission from AIP Publishing, 2019.

PMCRs can be designed based on manipulation of hotspots in nanostructured surfaces and thin films. As we know, the hotspots of Au nanobowl arrays are located on the edge of the Au nanobowl, as shown in Figure 7a. Due to the formation, distribution, and evolution of hotspots under the photoinduced effect of the enhanced EM field, the Au nanobowl arrays induce a photoreaction, leading to accelerated chemical reaction on defined positions. Interestingly, the size and position of the AgNPs are precisely controlled by polarized light and reaction time, as shown in Figure 7b–d. When using vertically circular polarized light incident to the surface of the Au nanobowl arrays, a six-axis symmetric patterned arrays of AgNPs growth is achieved. Furthermore, three-axis symmetric nanostructured arrays are obtained using linearly polarized oblique waves with a incidence angle of 50 degrees. The manipulation of hotspots can be used to accurately control a chemical reaction at the nanometer level, which has significant applications [41,42,43,44,45,46,47,48,53,55].

NSL-based nanostructured surfaces and thin films have also been focused on various fields, including superhydrophobicity [62], protein patterning [63], magnetization reversal [64], solar cells [65], light trapping enhancement [66], resonant optical transmission [31], flexible broadband antireflective coatings [67], flexibly tunable smart displays, and many others [68], which show more novel and interesting properties.

## 4. Conclusions and Outlook

In summary, various hybrid and even complex nanostructured surfaces and thin films can be designed and achieved by the combination of NSL, physical vapor deposition technique, etching, transfer, chemical reactions, or their combination steps. The formation, distribution, and evolution of hotspots can be manipulated by fabricating novel nanohoneycomb and SiO_2_−Ag nanocap arrays to control the enhancement of the EM field, which has potential applications in PEMS and PMCRs. In particular, detection of HCC based on SERS and controlling the growth locations of AgNPs are attracting more and more attention, which is expected to enhance the sensing performance. However, solving the defect formation during the self-assembly process for nanostructure-based devices is necessary in NSL technology. The notable result on defect-free PS colloid sphere arrays over a large area (36 wafers and 1 m^2^) has been demonstrated using the micropropulsive injection method to achieve high-throughput (6 × 6 wafers) periodic surface nanotexturing [58]. Recently, NSL-based nanostructured surfaces and thin films with functional materials have shown significant application in emerging magnetic skyrmion-based spintronic devices [69]. Thus, further functionalization of nanostructured surfaces and thin films with more ingenious designs are expected to allow for unprecedented versatility of NSL in a broad range of applications.
